# Deterioration of liver function and aging disturb sequential systemic therapy for unresectable hepatocellular carcinoma

**DOI:** 10.1038/s41598-022-21528-2

**Published:** 2022-10-11

**Authors:** Shigeo Shimose, Atsushi Hiraoka, Masatoshi Tanaka, Hideki Iwamoto, Takaaki Tanaka, Kazunori Noguchi, Hajime Aino, Taizo Yamaguchi, Satoshi Itano, Hideya Suga, Takashi Niizeki, Etsuko Moriyama, Tomotake Shirono, Yu Noda, Naoki Kamachi, Shusuke Okamura, Masahito Nakano, Takumi Kawaguchi, Ryoko Kuromatsu, Hironori Koga, Takuji Torimura

**Affiliations:** 1 Division of Gastroenterology, Department of Medicine, Kurume University School of Medicine, 67 Asahi-machi, Kurume, Fukuoka 830-0011 Japan; 2 Gastroenterology Center, Ehime Prefectural Central Hospital, Matsuyama, 790-0024 Japan; 3 Clinical Research Center, Yokokura Hospital, Miyama, Fukuoka 839-0295 Japan; 4 Department of Gastroenterology, Omuta City Hospital, Omuta, 836-0861 Japan; 5 Division of Gastroenterology, Department of Medicine, Social Insurance Tagawa Hospital, Tagawa, 826-0023 Japan; 6 Iwamoto Internal Medical Clinic, Kitakyusyu, 802-0832 Japan; 7 Department of Gastroenterology, Kurume Central Hospital, Kurume, 830-0001 Japan; 8 Department of Gastroenterology and Hepatology, Yanagawa Hospital, Yanagawa, 832-0077 Japan

**Keywords:** Cancer, Oncology

## Abstract

This study aimed to investigate the clinical characteristics of patients with unresectable hepatocellular carcinoma (HCC), who were eligible for sequential systemic therapy. We evaluated 365 patients with HCC who underwent systemic therapy after 2017. The overall survival (OS) was 13.7 months, 19.2 months, and 35.6 months in the first-line, second-line, and third-line or later therapy groups, respectively. Multivariate analysis revealed that the modified-albumin-bilirubin (m-ALBI) grade, macrovascular invasion, extrahepatic spread, discontinuation due to adverse events (AEs), and sequential therapy were independent factors for OS. At the end of each therapy, the ALBI score was significantly worse among patients with discontinuation due to AEs than among those without. The conversion rate to second-line and third-line therapy among patients with discontinuation due to AEs was significantly lower than that among patients without (30.4% vs. 69.2%, *p* < 0.001; 6.7% vs. 58.3%; *p* < 0.001, respectively). In the decision tree analysis, m-ALBI grade 1 or 2a and non-advanced age were selected splitting variables, respectively, for sequential systemic therapy. In conclusion, sequential therapy prolonged the OS of unresectable HCC. Additionally, good hepatic function and non-advanced age were clinically eligible characteristics for sequential systemic therapy.

## Introduction

The systemic therapy strategy for patients with unresectable hepatocellular carcinoma (HCC) begins with sorafenib (SORA)^1^ as the first-line therapy. For a decade, SORA remained the only approved molecular-targeted agent (MTA) for unresectable HCC, with patients showing a poor prognosis^2^. However, there has been a significant change in systemic therapy for patients with HCC in the last few years. There have been rapid developments in systemic therapy, with various MTAs including regorafenib (REGO)^3^, lenvatinib (LEN)^4^, ramucirumab (RAM)^5^, and cabozantinib (CAB)^6^. Moreover, atezolizumab plus bevacizumab (Atezo/Beva)^7^ as a combination therapy with an immune checkpoint inhibitor and an anti-angiogenic agent was approved in 2020, with further expansion of treatment strategies for patients with unresectable HCC.

Systemic therapy is the main strategy for patients with intermediate-stage HCC who are refractory or unsuitable for transarterial chemoembolization as well as for patients with advanced-stage HCC^8,9^. Sequential therapy involving switching across MTAs is currently the primary evidence-based treatment strategy^10^. Continuous approved MTA treatment significantly prolongs overall survival (OS) in patients with unresectable HCC^11,12^. Additionally, studies have reported the efficacy and safety of second-line treatment with MTAs after disease progression under treatment with Atezo/Beva^13^. Therefore, sequential systemic therapy is considered to prolong the prognosis for patients with unresectable HCC.

In the era of sequential therapy using various systemic therapies, we should first familiarize ourselves with the adverse events (AEs) associated with each systemic therapy^14,15^; further, careful management of AEs is necessary to avoid treatment discontinuation in patients receiving systemic therapy. Sequential therapy has been shown to prolong OS in patients with unresectable HCC^16,17^, with preserved hepatic function being an important factor for successful sequential therapy^16^. However, the relationship among preserved hepatic function, sequential therapy, and treatment discontinuation due to AEs remains unclear. Further, there is a need to clarify whether these factors influence sequential systemic therapy as well as to determine the eligibility criteria for sequential therapy.

This study aimed to investigate whether sequential systemic therapy improved OS in patients with unresectable HCC. Additionally, we aimed to evaluate the clinical characteristics of patients eligible for sequential systemic therapy.

## Results

### Patient characteristics

Table 1 summarizes the baseline characteristics of the included patients. The median age was 73 years; further, 289 (79.2%) patients were men. The etiology of liver disease was hepatitis C virus in 177 (48.5%) patients, and 18.6% of hepatitis C patients (33/177) obtained sustained virologic response. The m-ALBI grades 1, 2a, and 2b were observed in 149 (40.8%), 120 (32.9%), and 96 (26.3%) patients, respectively. The median tumor size was 31 mm; additionally, 174 (47.7%) patients were BCLC stage C. Extrahepatic spread and macrovascular invasion were observed in 129 (35.3%) and 50 (13.7%) patients, respectively. Regarding the first-line therapy, 194 (53.2%), 127 (34.8%), and 44 (12.0%) patients were treated using LEN, SORA, and Atezo/Beva, respectively. The median observation time was 15.7, 16.9, and 6.1 months in LEN, SORA, and Atezo/Beva, respectively. Moreover, 156, 35, 6, 4, and 1 patient transitioned to second-, third-, fourth-, fifth-, and sixth-line therapies, respectively (Table 1).

**Table 1:** Patient characteristics.

Characteristic	All patients
Number	365
Age (years)	73 (35–93)
Sex (male/female)	289/76
PS (0/1/2/3)	329/34/1/1
Etiology (HBV/HCV/Others)	56/177/132
AST (U/L)	37 (13–160)
ALT (U/L)	27 (4–201)
ALBI score [median (range)]	− 2.51 (− 3.62 to − 1.44)
m-ALBI grade (1/2a/2b)	149/120/96
Maximum nodule diameter (mm)	31 (12–190)
BCLC stage (B/C)	191/174
Macrovascular invasion (Yes/no)	50/315
Extrahepatic spread (Yes/no)	129/236
AFP (ng/mL)	44 (1–470,335)
DCP (mAU/mL)	432.5 (3.3–236,226)
Introduction of systemic therapy (LEN/SORA/Atezo/Beva)	194/127/44
Observation time, months	
LEN	15.7 (1.7–47.5)
SORA	16.9 (1.2–60.5)
Atezo/Beva	6.1 (1.4–12.3)
Transition to systemic treatment (second/third/fourth/fifth/sixth)	(156/35/6/4/1)

Data are expressed as median (range), or number. PS, performance status; HBV, hepatitis B virus; HCV, hepatitis C virus; AST, aspartate transaminase; ALT, alanine aminotransferase; m-ALBI, modified albumin-bilirubin; BCLC, Barcelona Clinic Liver Cancer; AFP, α-fetoprotein; DCP, des-γ-carboxy prothrombin; LEN, lenvatinib; SORA, sorafenib; Atezo, atezolizumab; Beva, bevacizumab.

### Evaluation of the therapeutic response to each first-line drugs

Therapeutic responses to each first-line drug are shown in Supplementary Fig. 1. The objective response rate (ORR) was observed in 36% of patients (16/44), 43% of patients (84/194), and 8% of patients (11/127) in the Atezo/Beva group, in the LEN group, and in the SORA group, respectively (Supplementary Fig. 1). On the other hand, the disease control rate was observed in 84% of patients (37/44), 79% of patients (154/194), and 44% of patients (56/127), respectively. Although there was no significant difference in the response between the Atezo/Beva group and the LEN group, there was a significant difference in the response between the SORA group and the other treatment groups (*p* = 0.001).

### Overall survival with each first-line drugs

The median survival time (MST) was not reached, 19.3 months, and 17.3 months among patients who received Atezo/Beva, LEN, and SORA, respectively (Supplementary Fig. 2). There was no difference in OS among the LEN, SORA, and Atezo/Beva groups as first-line therapy.

### The reasons for treatment discontinuation in the first-line and second-line therapy

The reasons for discontinuation observed during systemic therapy are shown in Table 2. Progressive disease was seen in 165 patients (54.0%), fatigue in 32 patients (10.4%), appetite loss in 22 patients (7.2%), and hepatic decompensation in 16 patients (5.1%) in the first-line therapy. On the other hand, progressive disease was seen in 68 patients (51.8%), fatigue in 10 patients (7.6%), appetite loss in 10 patients (7.6%) and hepatic decompensation in 12 patients (9.2%) in the second-line therapy (Table 2).

**Table 2:** The reasons for treatment discontinuation in the first-line and second-line therapy.

Factor	First-line therapy (n = 307) (%)	Second-line therapy (n = 131) (%)
**Progressive disease**	165 (54.0)	68 (51.8)
**Adverse events**	113 (36.7)	42 (32.1)
Fatigue	32 (10.4)	10 (7.6)
Appetite loss	22 (7.2)	10 (7.6)
Proteinuria	15 (4.9)	4 (3.1)
Liver disorder	13 (4.2)	6 (4.6)
Diarrhea	9 (2.9)	3 (2.3)
HFSR	8 (2.6)	5 (3.8)
Thrombocytopenia	5 (1.6)	1 (0.8)
Fever	4 (1.3)	2 (1.5)
Pneumonia	3 (0.9)	1 (0.8)
Skin disorders	2 (0.7)	0 (0.0)
**Hepatic decompensation**	16 (5.1)	12 (9.2)
Ascites	7 (2.3)	9 (6.9)
Encephalopathy	5 (1.6)	3 (2.3)
Hemorrhage	3 (0.9)	0 (0.0)
Jaundice	1 (0.3)	
Conversion	1 (0.3)	1 (0.8)
Others	12 (3.9)	8 (6.1)

*HFSR* hand-foot-syndrome-reaction.

### Univariate and multivariate analyses of factors for discontinuation of AEs

Multivariate analysis revealed that advanced age and modified-ALBI grade 2b were identified as independent factors associated with treatment discontinuation due to AEs (Supplementary Table 1).

### The difference in the reason for treatment discontinuation between the < 75 years and ≥ 75 years groups in the first-line and second-line therapy

The difference in the reason for treatment discontinuation between the < 75 years and ≥ 75 years groups are shown in Supplementary Tables 2 and 3.

The prevalence of discontinuation due to progressive disease was significantly higher in the < 75 years group compared to in the ≥ 75 years group in the first and second-line therapy, respectively. On the other hand, the prevalence of discontinuation due to fatigue was significantly higher in the ≥ 75 years group compared to in the < 75 years group in the first-line therapy (Supplementary Table 2). In addition, the prevalence of discontinuation due to appetite loss was significantly higher in the ≥ 75 years group compared to in the < 75 years group in the second-line therapy (Supplementary Table 3).

We also evaluated the baseline characteristics according to age (75 years) in patients with end first-line therapy. Regarding ALBI score, it was significantly different between the < 75 years group and ≥ 75 years group; however, there was no significant difference in the rate of initial dose reduction between the < 75 years and ≥ 75 years group in SORA treatment and LEN treatment (Supplementary Table 4). The cut-off age (< 75 years and ≥ 75 years) was based on the result of the data-mining analysis to determine the profiles associated with sequential therapy rates.

### Conversion rate to later-line sequential therapy and therapeutic agents

**Figure 1:**
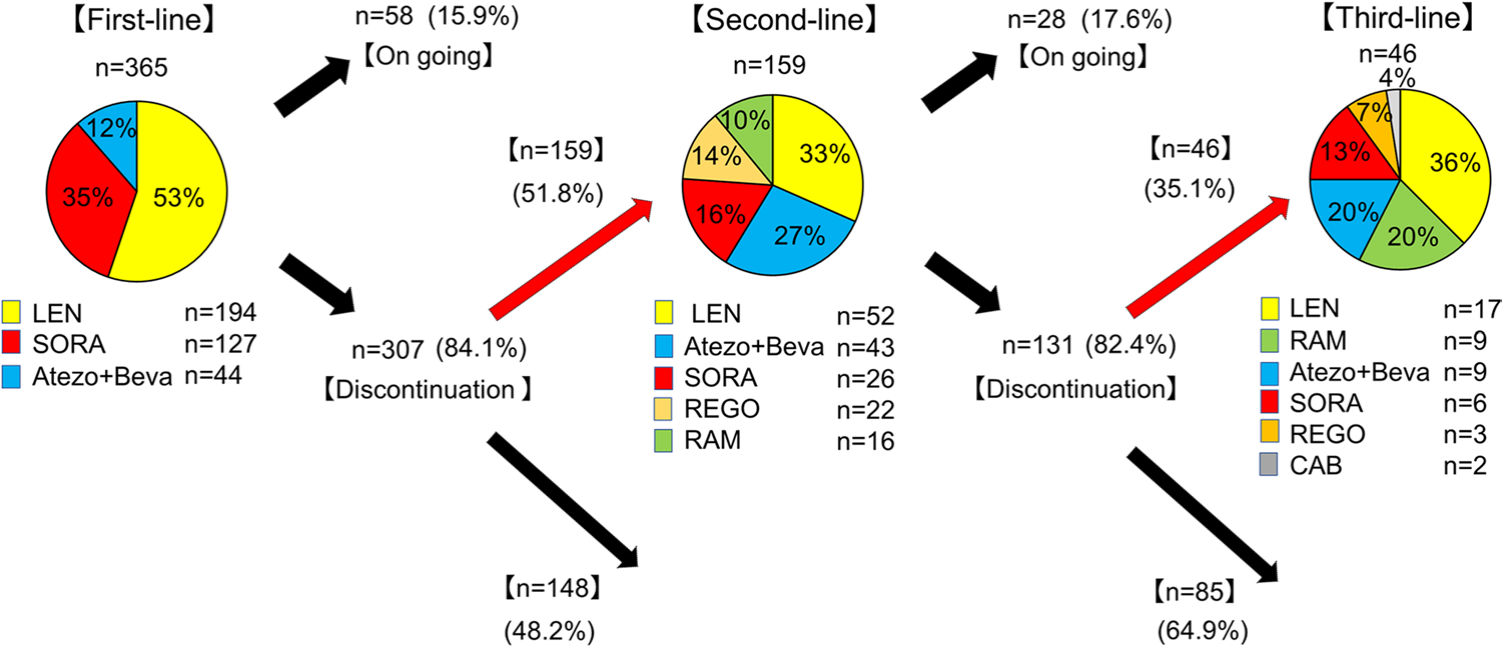
Conversion rate to later-line sequential therapy and therapeutic agents. The yellow, red, blue, orange, green, and gray blocks indicate LEN, SORA, atezolizumab plus bevacizumab, SORA, REGO, RAM, and CAB, respectively. Abbreviations: LEN, lenvatinib; SORA, sorafenib; REGO, regorafenib; RAM, ramucirumab; CAB, cabozantinib.

Figure 1 shows the conversion rate to later-line sequential MTA therapy. Among the included patients, 58 (15.9%) and 307 (84.1%) patients continued and discontinued first-line therapy, respectively. Among the patients who discontinued first-line therapy, 159 (51.8%) patients underwent second-line systemic therapy. LEN, Atezo/Beva, SORA, REGO, and RAM were used as second-line therapy in 52 (32.7%), 43 (27.0%), 26 (16.4%), 22 (13.8%), and 16 (10.1%) patients, respectively. Among the patients who received second-line therapy, 28 (17.6%) and 131 (82.4%) patients continued and discontinued treatment, respectively. Among the patients who discontinued second-line therapy, 46 (35.1%) patients underwent third-line systemic therapy. LEN, RAM, Atezo/Beva, SORA, REGO, and CAB were used as third-line therapy in 17 (36.9%), 9 (19.6%), 9 (19.6%), 6 (13.0%), 3 (6.5%), and 2 (4.4%) patients, respectively (Fig. 1). The most common sequential therapy until second-line and third-line therapy were SORA-LEN sequential therapy (27%:43/159) and SORA-REGO-LEN sequential therapy (34.8%:15/43), respectively.

### Relationship between discontinuation due to AEs and transition to the subsequent systemic therapy

Table 3 shows the rates of discontinuation of first- and second-line therapies due to AEs. There were 138 (44.9%) and 59 (45.0%) patients who discontinued first-line and second-line treatment, respectively, due to AEs (Table 3). The conversion rate to second-line and third-line therapies was significantly lower among patients with discontinuation due to AEs than among those without (30.4% vs. 69.2%, *p* < 0.001; 6.7% vs. 58.3%; *p* < 0.001, Table 3).

**Table 3:** Relationship between discontinuation due to AE and transition to next systemic therapy.

Variables	All patients	Discontinuation due to AE	No discontinuation due to AE	*p*
End of first-line therapy	307	138	169	
Transition to second-therapy (Yes/No)	159/148	42/96	117/52	< 0.001
Conversion rate to second-line therapy	51.8%	30.4%	69.2%	< 0.001
End of second-line therapy	131	59	72	
Transition to third-therapy (Yes/No)	46/85	4/55	42/30	< 0.001
Conversion rate to third-line therapy	35.1%	6.7%	58.3%	< 0.001

*AE* adverse event.

### Overall survival with systemic therapy according to first-, second-, and third-line or later therapies

**Figure 2:**
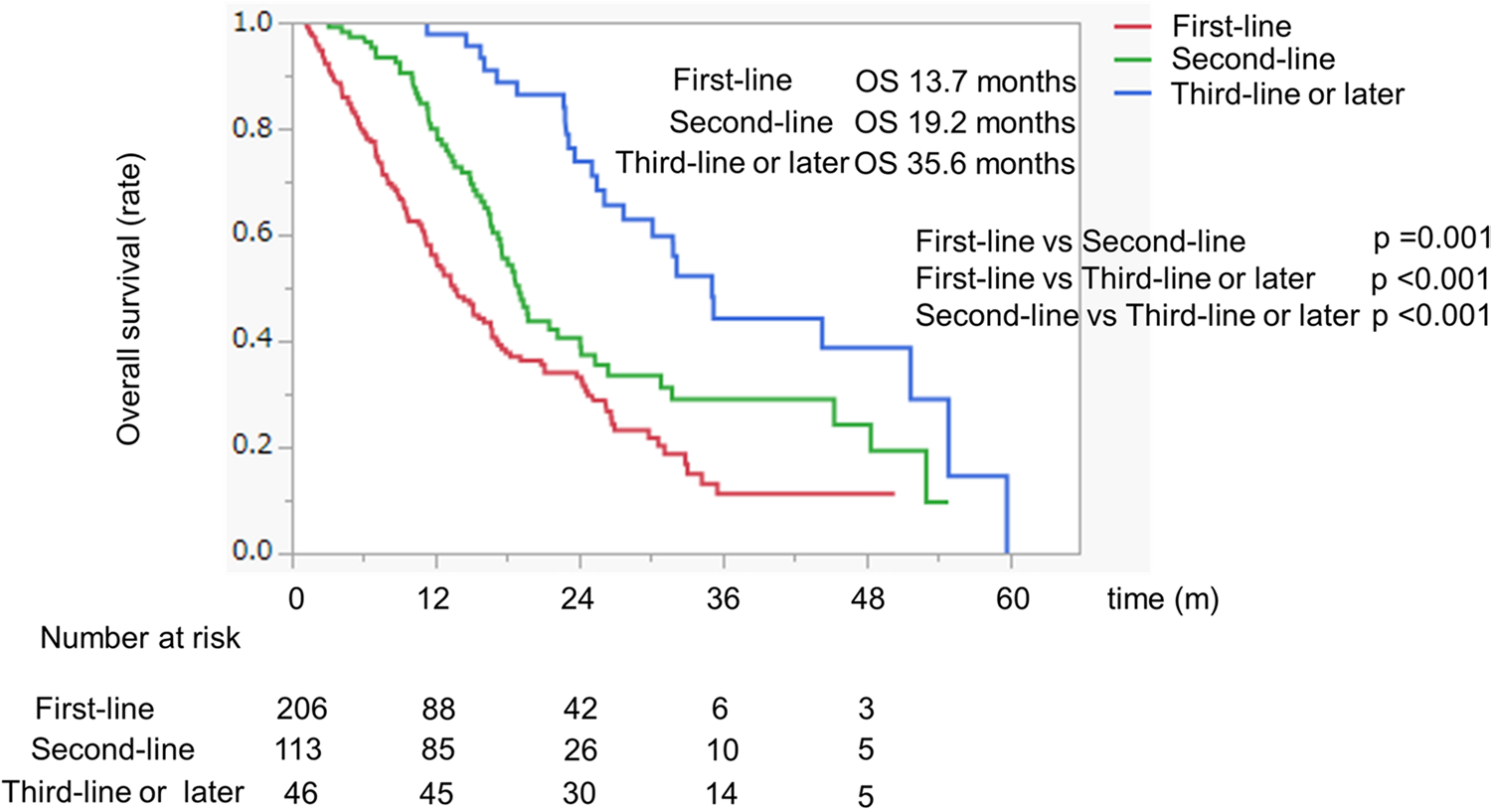
The overall survival of patients with HCC treated with systemic therapy. The red, green, and blue lines indicate the first-line, second-line, and third-line or later-line therapy groups, respectively. Abbreviations: HCC, hepatocellular carcinoma.

The median survival time (MST) was 13.7 months, 19.2 months, and 35.6 months among patients who received first-line, second-line, and third-line or later therapies, respectively (Fig. 2). In the baseline characteristics, younger age and better ALBI scores were detected in the group who could reatch to the third-line or later-line therapy, when compared to the first-line or second-line groups (Supplementary Table 5).

### ALBI score after first-, second-, and third-line or later therapies

**Figure 3:**
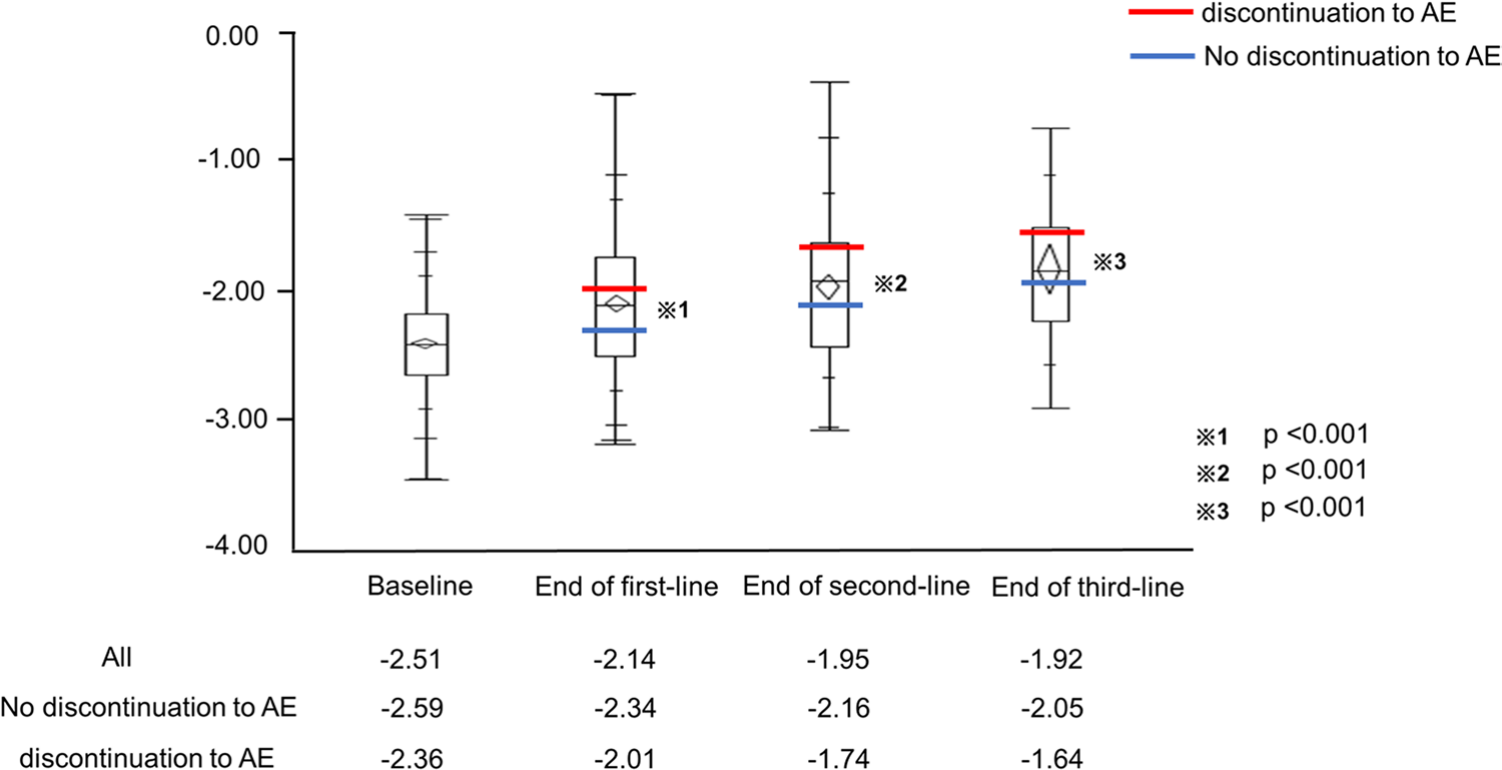
The albumin-bilirubin (ALBI) score over time in systemic therapy. The red and blue lines indicate the treatment discontinuation and no treatment discontinuation due to AEs, respectively. Abbreviations: AE, adverse event.

Figure 3 shows the changes in the ALBI score from baseline. The median ALBI scores at the end of first-line, second-line, and third-line treatment were − 2.14, − 1.95, and − 1.92, respectively. The ALBI score was significantly worse among patients who discontinued treatment due to AEs during the sequential systemic therapy than among patients who did not [(− 2.01 vs. − 2.34, *p* < 0.001: first-line therapy), (− 1.74 vs. − 2.16, *p* < 0.001: second-line therapy), (− 1.64 vs. − 2.05, *p* < 0.001: third-line therapy)].

### Decision tree analysis for transition to sequential therapy

**Figure 4:**
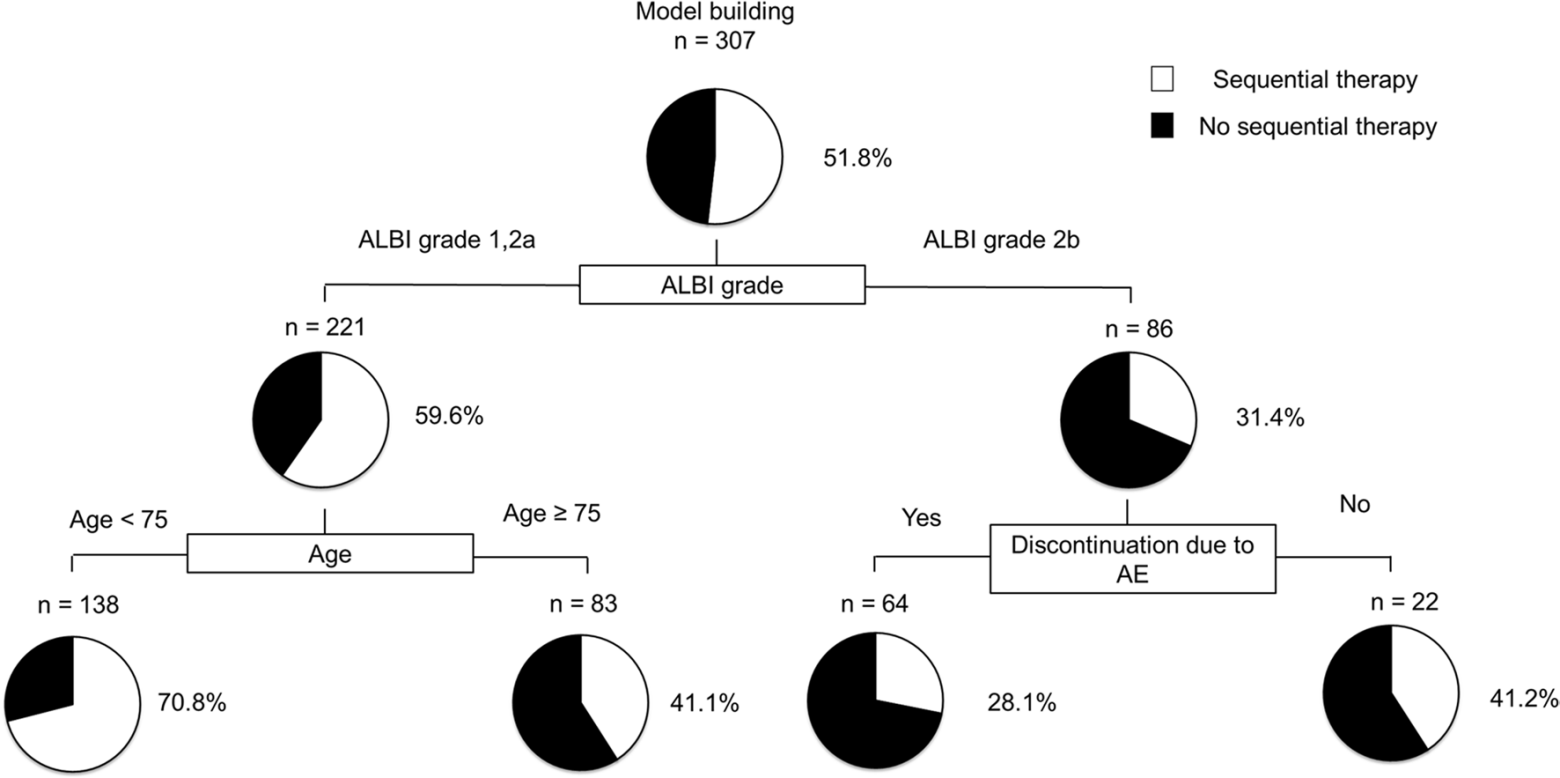
Profiles associated with sequential therapy. The pie graphs indicate the percentage of sequential therapy (white)/no sequential therapy (black) in each group.

We performed a decision tree analysis to determine the profiles associated with sequential therapy rates. It showed that the m-ALBI grade was the first splitting variable for sequential therapy rates. The sequential therapy rates were 31.4% and 59.6% in patients with m-ALBI grade 2b and m-ALBI grade 1 or 2a, respectively (Fig. 4). Among patients with m-ALBI grade 1 or 2a, the second splitting variable was age. The sequential therapy rate among patients with m-ALBI grade 1 or 2a (age < 75 years) was 70.8%. Contrastingly, the sequential therapy rate was 28.1% among patients with m-ALBI grade 2b who discontinued treatment due to AEs (Fig. 4).

The results of univariate and multivariate analyses for transition to sequential therapy are summarized in Supplementary Table 6. Age, modified-ALBI grade, and discontinuation due to AEs were identified as independent factors for transition to sequential therapy (Supplementary Table 6).

### Univariate and multivariate analyses of factors related to OS

Age, m-ALBI grade, maximum nodular diameter, macrovascular invasion, extrahepatic spread, AFP, DCP, discontinuation due to AEs, and post-progression treatment were included as variables in the univariate analysis. Multivariate analysis revealed that age, m-ALBI grade, macrovascular invasion, extrahepatic spread, discontinuation due to AEs, and sequential therapy were independent factors for OS (Table 4).

**Table 4:** Univariate and multivariate analyses of factors for OS.

Variable	Univariate analysis	Multivariate analysis		
	*p*-value	Odds ratio	95% CI	*p*-value
Age, < 75 versus ≥ 75	< 0.001	0.583	0.428–0.795	0.001
Sex, male versus female	0.212			
PS, < 1 versus ≥ 1	0.005	0.867	0.554–1.357	0.528
Etiology HBV versus HCV vs others	0.908			
m-ALBI grade, 1/2a versus 2b	< 0.001	0.468	0.336–0.650	< 0.001
Maximum nodule diameter (< 30 versus ≥ 30)	0.002	0.756	0.552–1.032	0.081
Macrovascular invasion (no/yes)	0.001	0.559	0.375–0.833	0.001
Extrahepatic spread (No/Yes)	0.001	0.638	0.399–0.889	0.002
AFP, < 400 versus ≥ 400 ng/mL	0.002	0.702	0.542–1.028	0.064
DCP, < 400 versus ≥ 400 mAU/mL	0.008	0.888	0.657–1.199	0.438
Discontinuation due to AE (+/−)	< 0.001	1.931	1.438–2.593	< 0.001
Sequential therapy (+/−)	< 0.001	0.494	0.366–0.667	< 0.001

*PS* performance status, *HBV* hepatitis B virus, *HCV* hepatitis C virus, *ALBI score* Albumin-bilirubin score, *BCLC* Barcelona Clinic Liver Cancer, *l AFP* α-fetoprotein, *DCP* des-γ-carboxy prothrombin, *AE* adverse event.

## Discussion

This study evaluated the efficacy of sequential systemic therapy and investigated the clinical characteristics of patients, who were eligible for sequential systemic therapy. Systemic sequential therapy improved the prognosis of patients with advanced HCC. Multivariate analysis revealed that age, m-ALBI grade 1 or 2a, macrovascular invasion, extrahepatic spread, treatment discontinuation due to reasons other than AE, and sequential therapy were independent factors for OS. Additionally, decision tree analysis revealed that m-ALBI grade 1 or 2a and non-advanced age were clinically eligible characteristics for sequential systemic therapy.

Sequential therapy using MTAs has been recently considered an effective strategy for unresectable HCC in real-world clinical conditions^11,18^. During the era of mono-MTA, the MST of patients treated with SORA was 10.7 months^1^. Currently, various systemic agents, including REGO, RAM, CAB, and Atez/Beva are available. In our study, transition to later-line therapy prolonged OS compared with monotherapy. Compared with second-line therapy, third-line or later therapy drastically improved OS, with an MST of 35.6 months. Yengohag et al. reported that the median OS in the sequential treatment of SORA-REGO was 22.2 months^19^. Additionally, SORA as a first-line sequential systemic therapy for patients with unresectable HCC was found to prolong OS^12^. In our study, patients with HCC who successfully transited to third-line or later-line therapies showed the best prognosis. However, enforcement of the clinical trials regarding sequential systemic therapy is practically difficult to perform, therefore data on what is the best sequential strategy for unresectable HCC is lacking. Recently, the Markov model estimated the survival of different sequential strategies for unresectable HCC patients^20^. This model provided a strong rationale to support ongoing trials evaluating second-line after first-line ATezo/Beva therapy. Therefore, using these simulation models will be very important to determine the strategy of sequential therapy in real-world practice in the future.

The ALBI score and hepatic function are significant prognostic factors for sequential therapy for HCC^16^. Pre-treatment liver function based on the ALBI score was associated with discontinuation of MTA therapy due to AEs^21–23^. Some previous studies reported that ALBI scores gradually declines by sequential systemic therapy using MTAs^11,24^. We found that the ALBI score was significantly worse among patients with discontinuation due to AEs than among those without at the end of each line treatment and the liver function in pre-treatment was poorer in patients with discontinuation due to AEs compared to those without (ALBI score was − 2.36, and − 2.59, respectively). Moreover, the rate of transition to the next systemic therapy was lower among patients with discontinuation due to AEs than among those without. Hence, sequential systemic therapy should be introduced at the good liver function. Furthermore, we should be careful with hepatic decompensation during systemic chemotherapy. Hepatic decompensation hampers the uptake of subsequent lines of systemic treatment and it is associated with poorer survival after discontinuation of systemic chemotherapy^25^. Hence, the optimal management of liver function could improve the overall survival of HCC patients through long-term preservation of liver function^26,27^. In fact, the median post-first-line therapy survival time (PFST) was 5.3 months, 9.3 months, and 15.6 months among patients who had hepatic decompensation, adverse event, and HCC progression, respectively (Supplementary Fig. 3). Although there was no significant difference in PFST between hepatic decompensation and adverse event (PFST 5.3 months vs. 9.3 months, *p* = 0.010), we must promptly recognize hepatic decompensation and adequately manage it to allow the patient to continue the treatments.

Our findings demonstrated that sequential therapy and treatment discontinuation due to reasons other than AEs were among the independent factors for prolonged OS. Post-treatment progression is strongly correlated with post-progression survival (PPS); further, improved PPS is the most important factor for prolonging OS^28^. Ando et al.^29^ reported that sequential therapy with MTAs could improve the prognosis of patients who discontinued first-line therapy; further, good liver function is a favorable factor related to eligibility for second-line therapy. In this study, the median PPS was 6.2 months, 13.6 months, and 26.9 months in the non-transition to the second-line group, transitioned to the second-line group, and transitioned to the third-line or later therapies group, respectively (Supplementary Fig. 4). Thus, improved PPS is the most important factor for prolonging OS.

Currently, it has been reorted thah OS was significantly shorter among patients with discontinuation due to AEs than among those without^30,31^. Generally, fatigue and appetite loss are common AEs that cause discontinuation of systemic therapy; additionally, these AEs significantly affect the worsening ALBI scores^15,22,32^ and are associated with shortened treatment duration. In fact, the change in ALBI score was significantly worse in patients with discontinuation of treatment due to fatigue and appetite loss than in patients with discontinuation of treatment due to reasons other than fatigue and appetite loss in this study [Fatigue; (from − 2.34 to − 1.89 vs. from − 2.53 to − 2.19, *p* < 0.001: first-line therapy, from − 2.06 to − 1.49 vs. from − 2.45 to − 2.01, *p* < 0.001: second-line therapy)], [Appetite loss; (from − 2.46 to − 2.19 vs. from − 2.49 to − 2.13, n.s: first-line therapy, from − 2.39 to − 1.93 vs. from − 2.41 to − 2.01, *p* = 0.03: second-line therapy)]. In addition, time is required to recover from discontinuation due to AEs, with the tumor enlarging during treatment interruption^33^. Therefore, it is important to avoid discontinuation of sequential systemic treatment due to AEs. However, this study enrolled more patients who received LEN treatment in initial systemic chemotherapy. It has been reported that appetite loss and fatigue lead to discontinuation of treatment in LEN treatment^15,33,34^. In patients with discontinuation of initial systemic chemotherapy, the development of fatigue > G2 and appetite loss > G2 was significantly higher in the LEN group than in the SORA or Atezo/Beva groups [Fatigue > G2; LEN (35.9%) vs. SORA (3.3%), *p* =  < 0.001, LEN (35.9%) vs. Atezo/Beva (10.5%), *p* = 0.015, appetite loss > G2; LEN (29.4%) vs. SORA (6.6%), *p* =  < 0.001, LEN (29.4%) vs. Atezo/Beva (0.0%), p =  < 0.001, respectively]. Although we cannot deny that the results in the study were affected by high ratio of patients who were initially treated with LEN, the study showed that which factors are important for administration of sequential systemic therapy. Thus, we consider that it is important to investigate the clinically eligible characteristics for sequential systemic therapy.

Additionally, advanced age is a critical factor that causes discontinuation of systemic therapy due to AEs^15^. This could be attributed to patients with advanced age being vulnerable to the toxicity of anti-cancer agents^35^. Although some previous reports revealed that MTA-monotherapy could be used safely in elderly patients with advanced HCC^36–38^, there are few reportson whether the impact of advanced age is how to influence in sequential systemic therapy. In this study, we found that the incidence of fatigue, appetite loss were significantly higher in the ≥ 75 years group compared to in the < 75 years group (*p* =  < 0.001) in the first-line, and second line therapy, respectively (Supplementary Tables 1 and 2). Moreover, we found that the incidence of progressive disease was siginicantly higher in the < 75 years group compared to in the ≥ 75 years group. We have previously reported that advanced age is associated with the discontinuation of systemic therapy due to severe AEs^15^. It suggests that patients with advanced age are vulnerable to the toxicity of anti-cancer agents ^35^. In contrast, patients with non-advanced age are tolerable to the toxicity of the drug. Therefore, prevalence of progressive disease was relatively higher in the < 75 years group. Although clinical trials mostly include patients of non-advanced age, in real-world clinical settings, numerous patients with advanced age are treated with systemic therapy. It is important to promptly detect symptoms of AE in patients with advanced age during sequential systemic treatment.

To prevent worsening liver function and discontinuation due to AEs, we should actively introduce the nursing and pharmaceutical department intervention for education regarding self-monitoring and AEs management, and telephone follow-up in every 2–4 weeks is also important in proper management^39^. Moreover, we recently reported a useful protocol for LEN involving a 5 days-on/2 days-off administration schedule (the weekends-off protocol)^40^. However, a robust strategy to prevent worsening liver function and discontinuation due to AEs has not been established yet. Thus, additional clinical trials and real-world evidence could facilitate the establishment of a strategy for systemic sequential therapy.

This study has several limitations. First, this was a retrospective study. Second, there was a selection bias in drug selection. Especially, the selection of first-line drugs contributes to having a great effect on sequential systemic therapy^41^. Third, second-line and later-line therapies were performed at the oncologist’s discretion. Fourth, the follow-up time of Atezo/Beva was short. Fifth, we did not evaluate the dose modification and relative dose intensity of MTAs, the time of occurrence of AE, and oesophageal varices. Sixth, the factors that occurred after starting therapies, such as sequential therapy used in the analysis cause bias for OS. Nonetheless, this is the first study to clarify the relationship between sequential systemic therapy and m-ALBI 1 or 2a or non-advanced age. Thus, it is necessary to accumulate clinical data and establish beneficial sequential systemic therapy^20^. Future studies are warranted to establish an evidence-based strategy for an improved order of sequential systemic therapy.

In conclusion, we demonstrated that sequential systemic therapy improved the prognosis of patients with unresectable HCC. Additionally, we showed that discontinuation due to AEs resulting from deterioration of liver function and advanced age could disturb sequential systemic therapy. Finally, good hepatic function and non-advanced age are characteristics of clinical eligibility for sequential systemic therapy.

## Material and methods

### Study design and patients recruitment

A total of 1003 consecutive patients with unresectable HCC underwent systemic therapy as a first-line treatment from 2009 to November 30, 2021 and were registered at eight independent institutions in Japan. The exclusion criteria were as follows: treatment with systemic therapy before 2017 (n = 562), Child–Pugh class B or C (n = 50), Barcelona Clinic Liver Cancer (BCLC) stage A (n = 13), lost to follow-up (n = 10), or clinical trial (n = 3). Accordingly, we included 365 patients (Supplement Fig. 5). The study was conducted in accordance with the Helsinki Declaration and was approved by the Ethical Committee of Kurume University School of Medicine (Approval Code: 21006). Informed consent was obtained using the opt-out approach.

### Diagnosis of HCC

HCC was diagnosed using a combination of following serum markers and imaging: AFP and DCP, and imaging procedures, including ultrasonography, enhanced CT and MRI scan*s.* Macrovascular invasion was radiologically diagnosed by enhanced CT or MRI. In the study, macrovascular invasion included invasion into the portal vein and hepatic vein.

### Treatment protocol and safety evaluation

Sequential systemic therapy was selected based on the evidence-based clinical practice guidelines for HCC of BCLC staging and treatment strategy^8,42^. Based on the industrial recommendations, the patients underwent first-line therapy comprising SORA (Nexval; Bayel Co., Ltd., Osaka, Japan), LEN (Eisai Co., Ltd, Tokyo, Japan), and Atezo/Beva (Chugai Pharmaceutical Co. LTD, Tokyo, Japan). SORA introduced as first-line therapy until LEN was approved in Japan. After LEN was approved in 2018, LEN introduced as first-line therapy. Moreover, since Atezo/Beva was approved, we introduced Atezo/Beva as first-line therapy. Depending on the approval time, the observation starting point of SORA started in 2017, because regorafenib was approved as second-line in Japan. Moreover, the observation starting point of LEN and Atezo/Beva was started in March 2018 and October 2020, respectively. Regarding contraindications of Atezo/Beva, we did not administer Atezo/Beva therapy in patients with autoimmune diseases. AEs were evaluated based on the National Cancer Institute Common Terminology Criteria for Adverse Events. Treatment was discontinued in case of development of any unacceptable or serious AE, or observation of clinical tumor progression. Systemic treatment using the second-line or subsequent therapies was performed depending on the physician’s decision.

### Assessment of hepatic functional reserve

Hepatic functional reserve was evaluated using the albumin-bilirubin (ALBI) score. ALBI scores were calculated as follows: [Log10 bilirubin level (μmol/L) × 0.66] + [albumin level (g/L) ×  − 0.085]^43^. The modified ALBI (m-ALBI) grade was defined based on the ALBI score as follows: m-ALBI grade 1, ≤ -2.60; m-ALBI grade 2a, > − 2.60 to ≤ − 2.27; m-ALBI grade 2b, >  − 2.27 to ≤ − 1.39; m-ALBI grade 3, > − 1.39^43^.

### Evaluation of treatment response and follow up

The therapeutic response of HCC was evaluated based on the modified Response Evaluation Criteria in Solid Tumors^44^ using computed tomography or magnetic resonance imaging at 4–6 weeks after the initial treatment. Subsequently, evaluation was performed at 3 months intervals until death or the study endpoint (November 31, 2021).

### Statistical analysis

All statistical analyses were performed using JMP Pro version 15 (SAS Institute Inc., Cary, NC). All data are presented as the number or median (range). OS was calculated using the Kaplan–Meier method and analyzed using the log-rank test or Bonferroni method^45^. Factors related to OS were evaluated through univariate and multivariate analyses using the Cox proportional hazards model. Additionally, we performed a decision tree analysis to identify factors related to the possibility of sequential therapy, as previously described^15^. Statistical significance was set at a two-tailed *p*-value < 0.05.

### Decision-tree algorithm

A decision-tree algorithm was constructed to reveal profiles associated with the sequential systemic therapy according to the instructions provided with the R software package as previously described^46^. The following variables were used for the decision-tree analysis for sequential systemic therapy: we used the same variables in the Cox proportional hazard model analysis.

### Ethics approval statement

This study was approved by the Ethical Committee of Kurume University School of Medicine (Approval Code: 21074) and was conducted according to the Helsinki Declaration.

## Supplementary Information

Supplementary Tables.

Supplementary Figure 1.

Supplementary Figure 2.

Supplementary Figure 3.

Supplementary Figure 4.

Supplementary Figure 5.

Supplementary Legends.

## Data Availability

The datasets used and/or analysed during the current study available from the corresponding author on reasonable request.

## Abbreviations

HCC: Hepatocellular carcinoma
SORA: Sorafenib
MTAs: Molecular-targeted agents
REGO: Regorafenib
LEN: Lenvatinib
RAM: Ramucirumab
CAB: Cabozantinib
Atezo/Beva: Atezolizumab plus bevacizumab
OS: Overall survival
AE: Adverse event
BCLC: Barcelona Clinic Liver Cancer
ALBI: Albumin-bilirubin
AFP: Alpha-fetoprotein
DCP: Des-γ-carboxy prothrombin
