# Supplementary material for: Deterioration of liver function and aging disturb sequential systemic therapy for unresectable hepatocellular carcinoma
Source: Sci Rep. 2022 Oct 11;12:17018. doi: 10.1038/s41598-022-21528-2 (PMC9554046; doi:10.1038/s41598-022-21528-2)
Supplement: Supplementary file 1 — Supplementary Tables. [file 41598_2022_21528_MOESM1_ESM.docx]

Supplementary Table 1. Univariate and multivariate analyses of factors for discontinuation of AEs

| Variable |  | Univariate analysis | Multivariate analysis | | | |
| --- | --- | --- | --- | --- | --- | --- |
|  | discontinuation due to AE  (Yes/No) | P-value | Odds ratio | 95% CI | | P-value |
| Age  <75  ≥75 | (43.9%/56.1%)  (57.6%/42.1%) | 0.001 | 0.633 | 0.413-0.972 | 0.036 | |
| Sex  male  female | (48.8%/51.2%)  (53.9%/46.1%) | 0.423 |  |  |  | |
| PS  <1  ≥1 | (48.6%/51.4%)  (61.1%/38.9%) | 0.153 |  |  |  | |
| Etiology of HCC  HBV  HCV  Others | (40.2%/59.8%)  (56.5%/43.5%)  (48.5%/51.5%) | 0.111 |  |  |  | |
| mALBI grade  1,2a  2b | (43.9%/56.1%)  (66.7%/33.3%) | <0.001 | 0.415 | 0.254-0.681 | <0.001 | |
| BCLC  B  C | (51.3%/48.7%)  (48.2%/51.8%) | 0.562 |  |  |  | |
| AFP  <400  ≥400 | (53.0%/47.0%)  (48.7%/51.3%) | 0.460 |  |  |  | |

Abbreviations: AE, adverse event; PS, performance status; HBV, hepatitis B virus; HCV, hepatitis C virus; ALBI score, albumin-bilirubin score; BCLC, Barcelona Clinic Liver Cancer; AFP, α-fetoprotein

Supplementary Table 2. The reasons for treatment discontinuation between < 75 years and ≥ 75 years in first-line therapy

| Factor | First-line therapy  (n=307) | Age < 75  (n=172) | Age ≥ 75  (n=135) | P |
| --- | --- | --- | --- | --- |
| Progression disease | 165 (54.0%) | 106 (61.6%) | 59 (43.7%) | 0.001 |
| Fatigue | 32 (10.4%) | 8 (4.6%) | 24 (17.8%) | <0.001 |
| Appetite loss | 22 (7.2%) | 11 (6.3%) | 11 (8.2%) | 0.554 |
| Proteinuria | 15 (4.9%) | 7 (4.0%) | 8 (6.0%) | 0.454 |
| Liver disorder | 13 (4.2%) | 10 (5.8%) | 3 (2.2%) | 0.120 |
| Diarrhea | 9 (2.9%) | 2 (1.2%) | 7 (5.2%) | 0.055 |
| HFSR | 8 (2.6%) | 5 (2.9%) | 3 (2.2%) | 0.708 |
| Ascites | 7 (2.3%) | 2 (1.2%) | 5 (3.7%) | 0.138 |
| Thrombocytopenia | 5 (1.6%) | 2 (1.2%) | 3 (2.2%) | 0.466 |
| Encephalopathy | 5 (1.6%) | 2 (1.2%) | 3 (2.2%) | 0.466 |
| Fever | 4 (1.3%) | 2 (1.2%) | 2 (1.5%) | 0.806 |
| pneumoniae | 3 (0.9%) | 2 (1.2%) | 1 (0.7%) | 0.709 |
| Hemorrhage | 3 (0.9%) | 2 (1.2%) | 1 (0.7%) | 0.709 |
| Skin disorders | 2 (0.7%) | 2 (1.2%) | 0 (0.0%) | 0.208 |
| Conversion | 1 (0.3%) | 1 (0.6%) | 0 (0.0%) | 0.374 |
| Others | 13 (4.2%) | 8 (4.6%) | 5 (3.7%) | 0.682 |

HFSR, hand-foot-syndrome-reaction

Supplementary Table 3. The reason for treatment discontinuation between < 75 years and ≥ 75 years in second-line therapy

| Factor | Second-line therapy  (n=131) | Age < 75  (n=85) | Age ≥ 75  (n=46) | P |
| --- | --- | --- | --- | --- |
| Progression disease | 68 (51.9%) | 52 (61.2%) | 16 (34.9%) | <0.001 |
| Fatigue | 10 (7.6%) | 5 (5.9%) | 5 (10.8%) | 0.304 |
| Appetite loss | 10 (7.6%) | 3 (3.5%) | 7 (15.2%) | 0.016 |
| Proteinuria | 4 (3.1%) | 2 (2.4%) | 2 (4.3%) | 0.526 |
| Liver disorder | 6 (4.6%) | 2 (2.4%) | 4 (8.7%) | 0.091 |
| Diarrhea | 3 (2.3%) | 1 (1.1%) | 2 (4.3%) | 0.246 |
| HFSR | 5 (3.8%) | 4 (4.7%) | 1 (2.2%) | 0.470 |
| Ascites | 9 (6.9%) | 5 (5.9%) | 4 (8.7%) | 0.543 |
| Thrombocytopenia | 1 (0.8%) | 1 (1.1%) | 0 (0.0%) | 0.460 |
| Encephalopathy | 3 (2.3%) | 2 (2.4%) | 1 (2.2%) | 0.947 |
| Fever | 2 (1.5%) | 2 (2.4%) | 0 (0.0%) | 0.294 |
| pneumoniae | 1 (0.8%) | 1 (1.1%) | 0 (0.0%) | 0.462 |
| Hemorrhage | 0 (0.0%) | 0 (0.0%) | 0 (0.0%) | n.s |
| Skin disorders | 0 (0.0%) | 0 (0.0%) | 0 (0.0%) | n.s |
| Conversion | 1 (0.8%) | 0 (0.0%) | 1 (2.2%) | 0.172 |
| Others | 8 (6.0%) | 5 (5.9%) | 3 (6.5%) | 0.884 |

HFSR, hand-foot-syndrome-reaction

Supplementary Table 4. Baseline characteristics according to age in patients with end first-line therapy

| Characteristic | Age < 75  (n=172) | Age ≥ 75  (n=135) | P-value |
| --- | --- | --- | --- |
| Sex (female/male) | 28/144 | 33/102 | 0.076 |
| PS (0/1/2/3) | 160/12/0 | 115/19/1 | 0.521 |
| Etiology (HBV/HCV/Others) | 42/73/57 | 7/80/48 | 0.001 |
| ALBI score  median (range) | -2.56  (-3.62- -1.48) | -2.39  (-3.29- -1.44) | 0.001 |
| m-ALBI grade, 1/2a/2b | 80/58/34 | 43/40/52 | 0.005 |
| BCLC stage (B/C) | 75/97 | 77/58 | 0.019 |
| Macrovascular invasion  (Yes/No) | 34/138 | 13/122 | 0.012 |
| Extrahepatic spread  (Yes/No) | 69/103 | 44/91 | 0.174 |
| Initial dose  LEN (4 mg/8 mg/12 mg)  SORA (200 mg/400 mg/600 mg/800 mg) | 2/49/37  3/53/1/14 | 5/60/14  4/37/1/8 |  |
| Dose reduction (Yes/No)  LEN (Yes/No)  SORA (Yes/No)  Atezo/Beva (Yes/No) | 9/79 (10.2%)  57/14 (80.2%)  0/13 (0.0%) | 9/70 (11.3%)  44/6 (88.0%)  0/6 (0.0%) | 0.808  0.260  n.s |
| AFP, median (range), ng/mL | 68.7  (1.3-177,630) | 55.5  (1.0-470,335) | 0.120 |
| DCP, median (range), mAU/mL | 333.0  (12.0-236,226) | 180.0  (3.3-180,779) | 0.061 |

Data are expressed as median (range), or number. PS, performance status; HBV, hepatitis B virus; HCV, hepatitis C virus; AST, aspartate transaminase; ALT, alanine aminotransferase; m-ALBI, modified albumin-bilirubin; BCLC, Barcelona Clinic Liver Cancer; LEN, lenvatinib; SORA, sorafenib; Atezo, atezolizumab; Beva, bevacizumab; AFP, α-fetoprotein; DCP, des-γ-carboxy prothrombin

Supplementary Table 5. Baseline patient characteristics

| Characteristic | End of first-line  (n=206) | End of second-line  (n=113) | End of third-line or later  (n=46) | P-value |
| --- | --- | --- | --- | --- |
| Age (years) | 75 (44-93) | 72 (35-89) | 68 (47-85) | <0.001 |
| Sex (female/male) | 51/155 | 21/92 | 4/42 | 0.061 |
| PS | 184/20/1/1 | 101/12/0/0 | 44/2/0/0 | 0.647 |
| Etiology (HBV/HCV/Others) | 25/104/77 | 20/55/38 | 11/18/17 | 0.259 |
| ALBI score  median (range) | -2.44  (-3.36- -1.44) | -2.55  (-3.61- -1.57) | -2.61  (-3.62- -1.72) | 0.001 |
| m-ALBI grade, 1/2a/2b | 73/64/69 | 51/41/21 | 25/15/6 | 0.005 |
| BCLC stage (B/C) | 104/102 | 65/48 | 22/24 | 0.390 |
| Macrovascular invasion  (Yes/No) | 33/173 | 10/103 | 7/39 | 0.173 |
| Extrahepatic spread  (Yes/No) | 74/132 | 41/72 | 14/32 | 0.752 |
| AFP, median (range), ng/ml | 34.1  (1.2-279,663) | 73.6  (1.0-470,335) | 24.8  (2.3-156,228) | 0.836 |
| DCP, median (range), mAU/ml | 199.0  (3.3-236,226) | 211.0  (12.0-127,950) | 394.5  (15.0-27,450) | 0.691 |

Data are expressed as median (range), or number. PS, performance status; HBV, hepatitis B virus; HCV, hepatitis C virus; m-ALBI, modified albumin-bilirubin; BCLC, Barcelona Clinic Liver Cancer; AFP, α-fetoprotein; DCP, des-γ-carboxy prothrombin

Supplementary Table 6. Univariate and multivariate analyses of factors for sequential therapy

| Variable |  | Univariate analysis | Multivariate analysis | | | |
| --- | --- | --- | --- | --- | --- | --- |
|  | Sequential therapy  (Yes/No) | P-value | Odds ratio | 95% CI | | P-value |
| Age  <75  ≥75 | (62.8%/37.2%)  (37.7%/62.3%) | <0.001 | 0.456 | 0.274-0.758 | 0.002 | |
| Sex  male  female | (54.5%/45.5%)  (40.9%/59.1%) | 0.055 |  |  |  | |
| PS  <1  ≥1 | (52.7%/47.2%)  (43.8%/56.2%) | 0.336 |  |  |  | |
| Etiology of HCC  HBV  HCV  Others | (63.2%/36.7%)  (47.1%/52.3%)  (52.4%/47.6%) | 0.168 |  |  |  | |
| m-ALBI grade  1, 2a  2b | (59.8%/40.2%)  (31.4%/68.6%) | <0.001 | 0.467 | 0.261-0.836 | 0.010 | |
| BCLC  B  C | (57.2%/42.8%)  (46.5%/53.5%) | 0.058 |  |  |  | |
| AFP  <400  ≥400 | (51.2%/48.8%)  (53.3%/46.7%) | 0.729 |  |  |  | |
| Discontinuation due to AE  Yes  No | (30.4%/69.7%)  (69.3%/30.7%) | <0.001 | 4.220 | 2.541-7.001 | <0.001 | |

Abbreviations: PS, performance status; HBV, hepatitis B virus; HCV, hepatitis C virus; ALBI score, Albumin-bilirubin score; BCLC, Barcelona Clinic Liver Cancer; l AFP, α-fetoprotein; AE, adverse event
