# Supplementary figures and images for: Deterioration of liver function and aging disturb sequential systemic therapy for unresectable hepatocellular carcinoma
Source: Sci Rep. 2022 Oct 11;12:17018. doi: 10.1038/s41598-022-21528-2 (PMC9554046; doi:10.1038/s41598-022-21528-2)

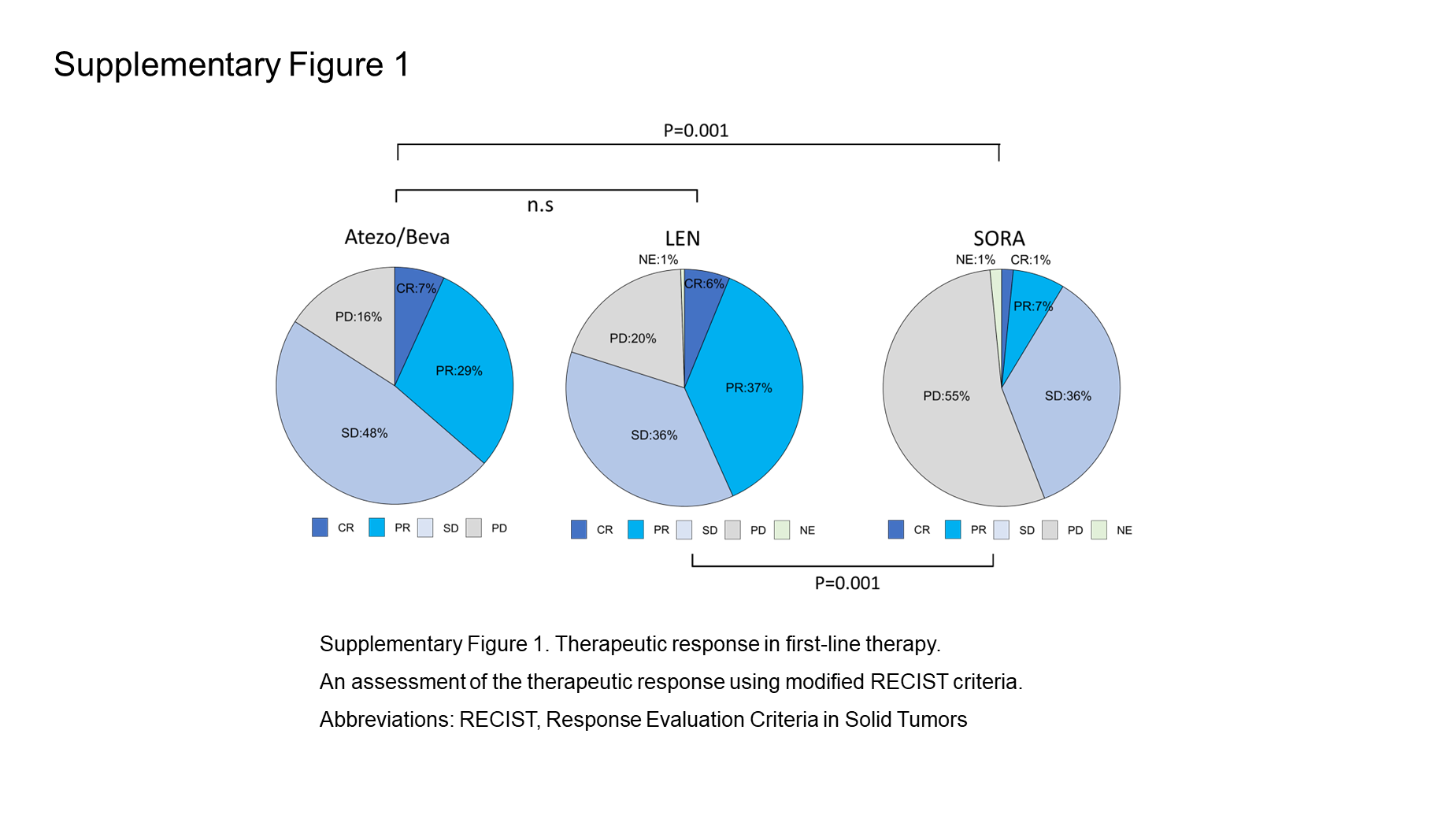

Supplement: Supplementary file 2 — Supplementary Figure 1. [file 41598_2022_21528_MOESM2_ESM.tif]

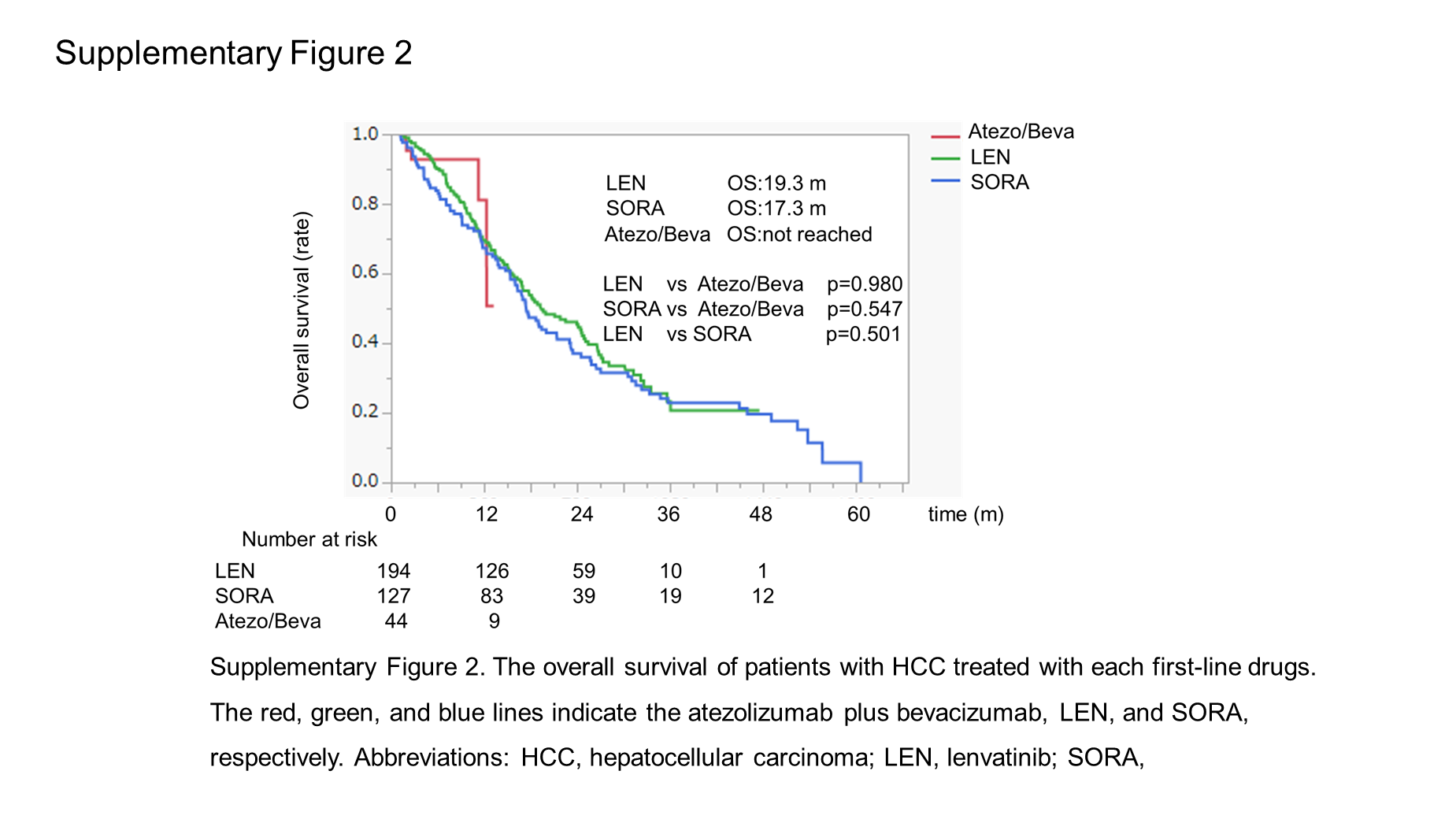

Supplement: Supplementary file 3 — Supplementary Figure 2. [file 41598_2022_21528_MOESM3_ESM.tif]

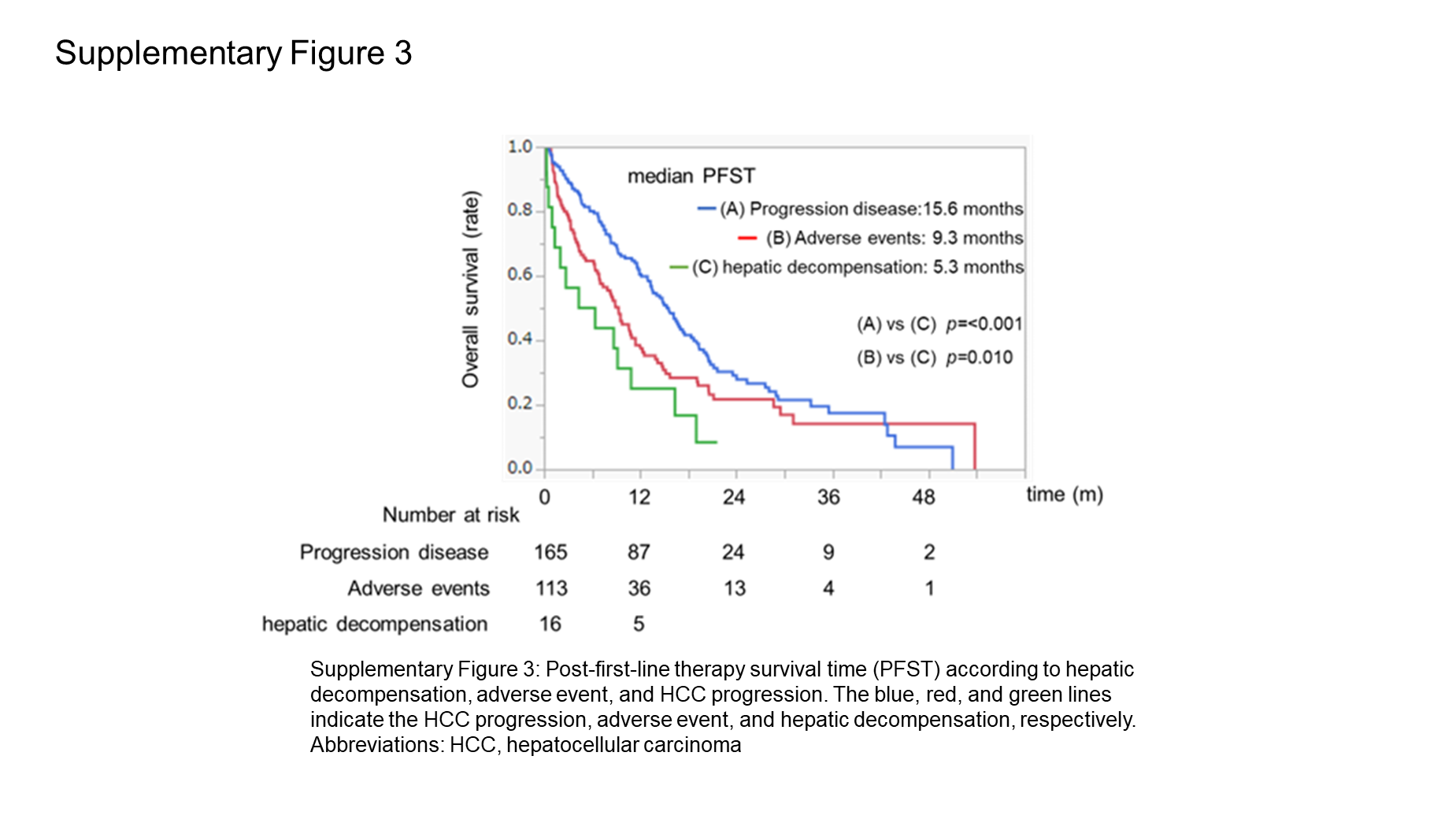

Supplement: Supplementary file 4 — Supplementary Figure 3. [file 41598_2022_21528_MOESM4_ESM.tif]

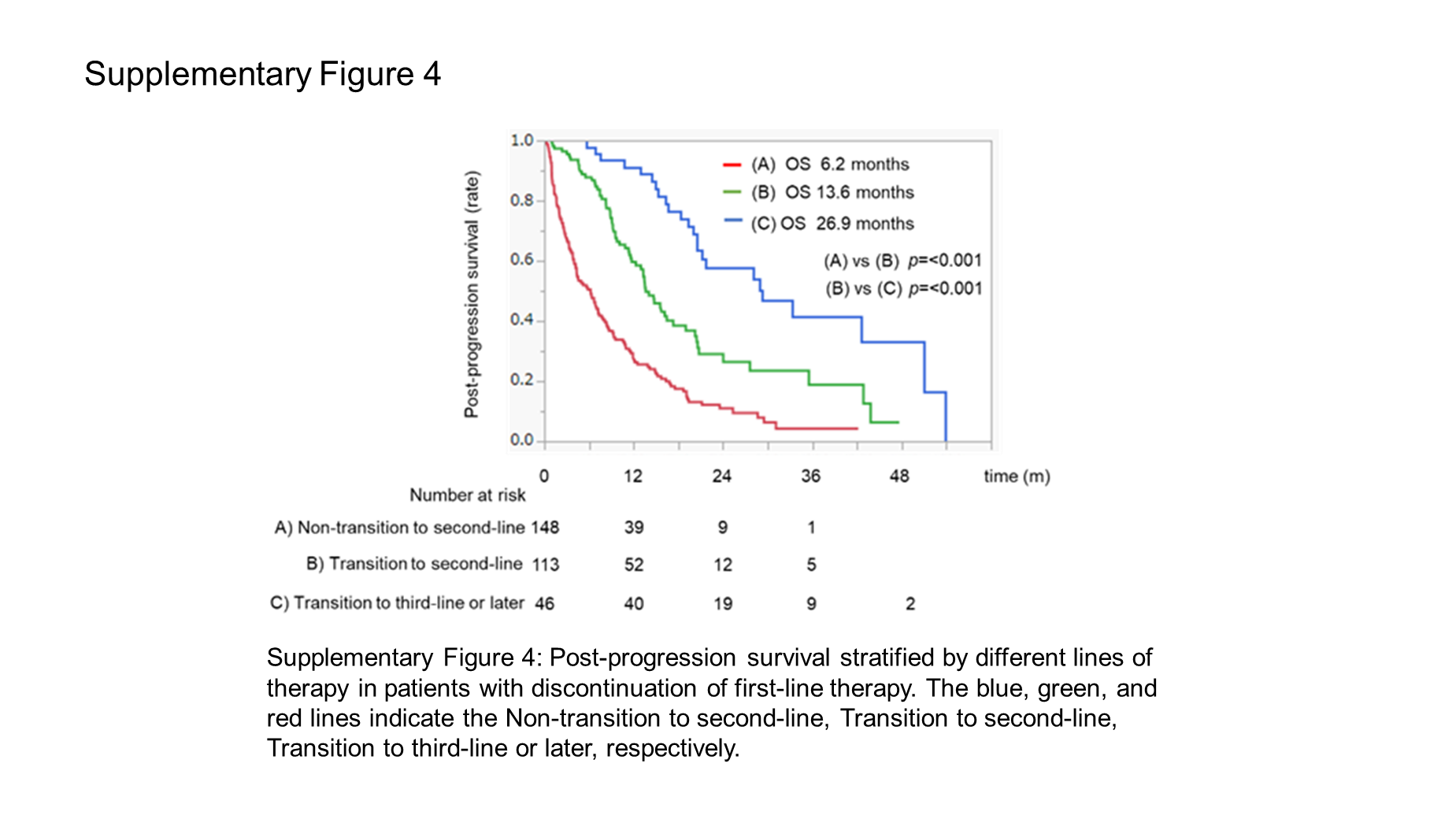

Supplement: Supplementary file 5 — Supplementary Figure 4. [file 41598_2022_21528_MOESM5_ESM.tif]

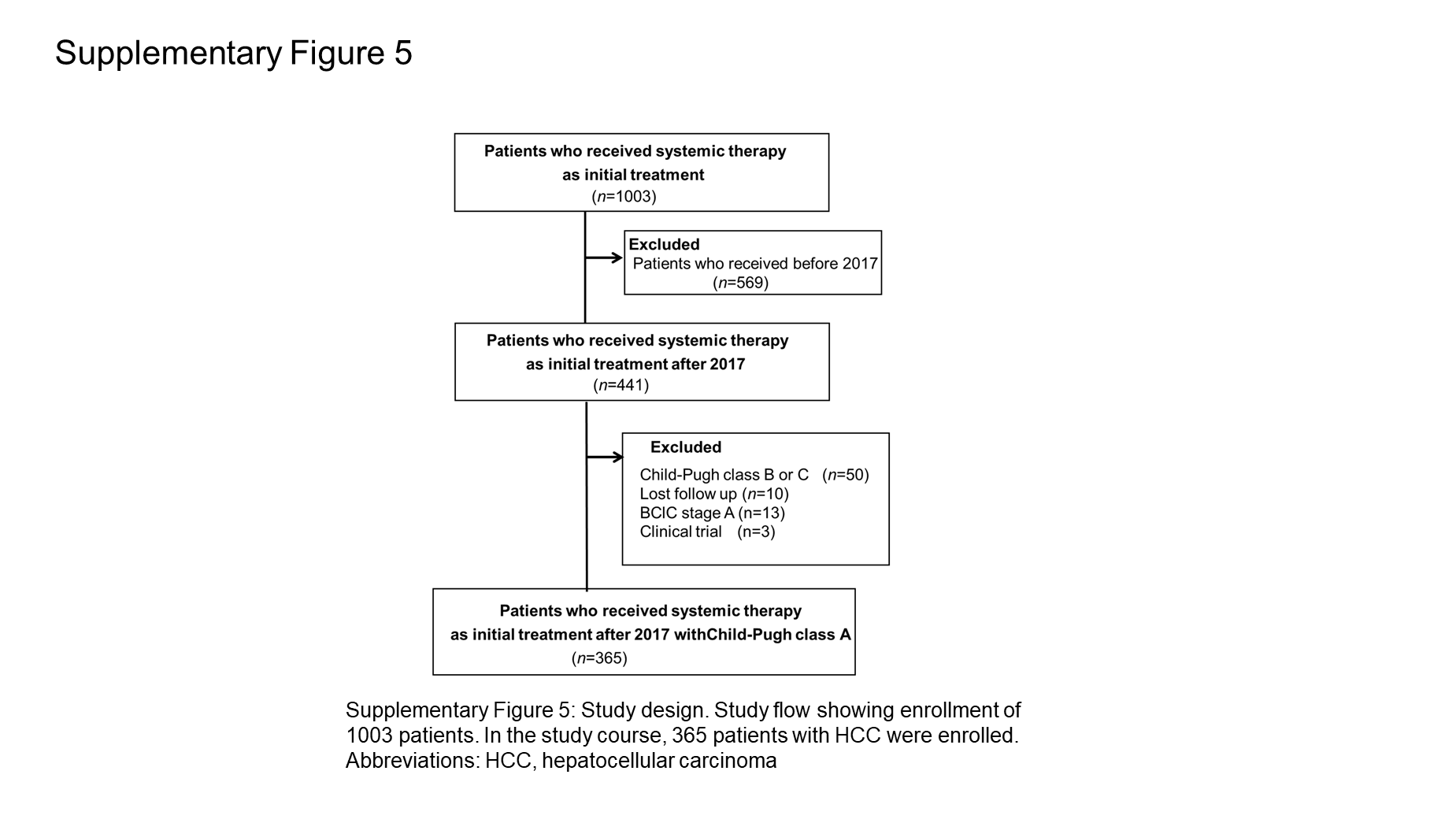

Supplement: Supplementary file 6 — Supplementary Figure 5. [file 41598_2022_21528_MOESM6_ESM.tif]
