# Supplementary material for: Deterioration of liver function and aging disturb sequential systemic therapy for unresectable hepatocellular carcinoma
Source: Sci Rep. 2022 Oct 11;12:17018. doi: 10.1038/s41598-022-21528-2 (PMC9554046; doi:10.1038/s41598-022-21528-2)
Supplement: Supplementary file 7 — Supplementary Legends. [file 41598_2022_21528_MOESM7_ESM.docx]

**Supplementary Figure legends**

Supplementary Figure 1. Therapeutic response in first-line therapy. An assessment of the therapeutic response using modified RECIST criteria.

Abbreviations: RECIST, Response Evaluation Criteria in Solid Tumors

Supplementary Figure 2. The overall survival of patients with HCC treated with each first-line drugs. The red, green, and blue lines indicate the atezolizumab plus bevacizumab, LEN, and SORA, respectively.

Abbreviations: HCC, hepatocellular carcinoma; LEN, lenvatinib; SORA,

Supplementary Figure 3: Post-first-line therapy survival time according to hepatic decompensation, adverse event, and HCC progression. The blue, red, and green lines indicate the HCC progression, adverse event, and hepatic decompensation, respectively.

Abbreviations: HCC, hepatocellular carcinoma

Supplementary Figure 4: Post-progression survival stratified by different lines of therapy in patients with discontinuation of first-line therapy. The blue, green, and red lines indicate the Non-transition to second-line, Transition to second-line, Transition to third-line or later, respectively.

Supplementary Figure 5: Study design. Study flow showing enrollment of 1003 patients. In the study course, 365 patients with HCC were enrolled.

Abbreviations: HCC, hepatocellular carcinoma
